# Effect of Service Temperature on the Mechanical and Fatigue Behaviour of Metal–Polymer Friction Stir Composite Joints

**DOI:** 10.3390/polym17101366

**Published:** 2025-05-16

**Authors:** Arménio N. Correia, Rodrigo J. Coelho, Daniel F. O. Braga, Mafalda Guedes, Ricardo Baptista, Virgínia Infante

**Affiliations:** 1 Instituto Superior Técnico, Universidade de Lisboa, Av. Rovisco Pais, 1049-001 Lisboa, Portugal; 2 INEGI, Faculdade de Engenharia, Universidade do Porto, Rua Dr. Roberto Frias, 400, 4200-465 Porto, Portugal; 3 UnIRE, ISEL, Instituto Politécnico de Lisboa, Av. Conselheiro Emídio Navarro 1, 1959-007 Lisboa, Portugal; 4 CeFEMA-LaPMET, Instituto Superior Técnico, Universidade de Lisboa, Av. Rovisco Pais, 1049-001 Lisboa, Portugal; 5 LAETA, IDMEC, Instituto Superior Técnico, Universidade de Lisboa, Av. Rovisco Pais, 1049-001 Lisboa, Portugal

**Keywords:** composite joints, friction stir joining, joint strength, fatigue strength, service temperature

## Abstract

This study investigates the mechanical and fatigue behaviour of friction stir composite joints fabricated from an aluminum alloy (AA6082-T6) and a glass fibre-reinforced polymer (Noryl^®^ GFN2) under different service temperature conditions. The joints were tested under both quasi-static and cyclic loading at three different temperatures (23, 75, and 130 °C). Fracture surfaces were analyzed, and the probabilistic S–N curves were derived using Weibull distribution. Results indicated that increasing the service temperature caused a non-linear decrease in both the quasi-static and fatigue strength of the joints. Compared to room temperature, joints tested at 75 °C and 130 °C showed a 10% and 50% reduction in average tensile strength, respectively. The highest fatigue strength occurred at 23 °C, while the lowest was at 130 °C, in line with the quasi-static results. Fatigue stress-life plots displayed a semi-logarithmic nature, with lives ranging from 10^2^ to 10^5^ cycles for stress amplitudes between 7.7 and 22.2 MPa at 23 °C, 7.2 to 19.8 MPa at 75 °C, and 6.2 to 13.5 MPa at 130 °C. The joints’ failure occurred in the polymeric base material close to joints’ interface, highlighting the critical role of the polymer in limiting joints’ performance, as confirmed by thermal and scanning electron microscopy analyses.

## 1. Introduction

The increasingly tight constrains of the transport sector regarding safety, lightweight, and sustainable design have been pushing academy and industry towards the development of innovative materials and disruptive technologies that enable their efficient bonding. In this context, friction stir welding (FSW) emerged as an efficient approach to the challenge of metal/polymer joining.

FSW is a solid-state technology, relying on a non-consumable tool that rotates and traverses along the joint line. The energy input is purely mechanical, resulting from friction heat generated between tool and workpiece surfaces [1]. As the materials undergo severe plastic strain, heat energy is released, stacking to the frictional one and further increasing the processing temperature within the joining area [2,3,4]. Typically, the material softens without reaching its melting point [1], and is transferred and forged by the tool to produce a weld between the two workpieces. Advantages of FSW include cost-effectiveness; high productivity and possibility for continuous welding (it is an automated process); simple joint design and no joint preparation; no filler materials required; very little thermal stress or distortion; environment friendly (absence of arc, fumes, radiation or noise) [5,6,7,8,9]. Overall, joint quality is consistently good, resulting in high fatigue strength and better-preserved mechanical properties compared to arc welding, and can be used also for alloys that are crack sensitive when welded via conventional fusion processes [1].

FSW has been used to join several materials, including some which are difficult to join via conventional fusion-based processes [2]. A range of similar and dissimilar metals (importantly including aluminum and its alloys), metal–matrix composites, and polymer–matrix composites can be friction stir joined to produce high-quality joints [1,2,4]. In what refers to combining metals with polymer-based materials, the most widely used technologies are mechanical fastening and adhesive bonding, yet with significant limitations [10]. While mechanical fastening requires an increased weight to compensate the stress concentration around the fastening holes, adhesive bonding mechanical performance may be severely jeopardized by in-service temperature and moisture. Table 1 displays a list of successful examples of metal–polymer friction stir joints reported in the literature, where most of them are produced in overlap configuration. This technical option results predominantly from the technological limitations imposed by lack of miscibility between metals and polymers. Rather than metallurgical joining, friction stir composite joints rely on mechanical interlocking and interfacial bonding, as primary binding mechanisms [7,11,12,13,14,15].

Additionally, joints’ mechanical strength, components, and structures reliability throughout service life is mandatory, withstanding cyclic loading without unforeseen failure. The wider use of friction stir joining in metal/polymer bonding is currently hindered by the need to gain further control upon static strength of the joints, and strength under cyclic loading conditions. Only few studies report the effect of processing and service parameters upon the performance of friction stir welded joints. Pinto et al. [26] assessed the effect of the loading temperature (room temperature and 150 °C) and stress ratio (0.05 and 0.5) over the number of cycles until failure on friction stir welded AA7xxx aluminum alloy in butt joint configuration. The quasi-static tensile tests revealed that the fabricated joints had similar UTS values when loaded under different temperatures, showing an average joint efficiency of 64%, yet with a noteworthy increase in ductility at 150 °C. The fatigue life of the specimens loaded at 150 °C was significantly decreased, in line with the quasi-static decrease, when compared to fatigue tests conducted at a similar stress range and the same stress ratio, at room temperature. It was also seen that the lower stress ratio also had a negative impact on fatigue life, either at room temperature or 150 °C.

Guster et al. [27] tested the fatigue behaviour of PA 6T/6I-GF50, a 50 wt.% of glass-fibres reinforced Polyamide, with a constant stress ratio of 0.1 and three levels of temperature (23, 80, and 120 °C). The test results displayed a decreasing fatigue strength with an increase in loading temperature, regardless of the applied stress amplitude. Mortazavian [28] assessed the fatigue behaviour of two glass fibre reinforced polymers, PBT and PA6 with 30 and 35 wt.% short glass fibre, respectively, under several loading conditions. The fatigue tests were carried out at temperatures of −40, 23, and 125 °C, exploring the associated effects under the stress ratios of −1.0, 0.1, and 0.3, and frequencies between 0.25 and 4 Hz. It was observed that there was a significant effect of temperature on fatigue behaviour on both materials in which, the −40 °C had a beneficial effect while increasing the temperature had a detrimental effect on fatigue life, as compared to room temperature behaviour. Moreover, a significant decrease in fatigue strength was found across the tensile mean stress range for both materials at all test temperatures, with more pronounced impact on the low cycle fatigue regime, as compared to the high cycle one. The obtained results are in line and support the results obtained by Guster et al. [27].

Ogawa et al. [24] studied the effect of welding time on static and fatigue strength of dissimilar friction stir spot welded joints between an aluminum alloy and a carbon fibre reinforced (CFR) polymer. It was found that both static and fatigue strength of the fabricated joints improved with an increased processing time. This behaviour was justified with the enlarged joining region and increased amount of molten resin melting. All the specimens failed close to the joining region, yet with different nucleation sites, namely, crack on the aluminum alloy clamped side, crack on the polymer clamped side, crack from interfacial pores, and crack from the molten resin layer. Lately, Ogawa et al. [29] also assessed how the clamping system affects the fatigue properties of dissimilar friction stir spot welded joints between an aluminum alloy and a CFR polymer. To that end, two clamping systems were used during the joining process—(i) constraint jig, and (ii) groove jig. The authors observed that the joints fabricated with a groove jig (the one that imposes less amount of fixture constrain) displayed the higher tensile and fatigue strength.

From the literature review, it is possible to assert that, to the best knowledge of the authors, no research work over the effects of service temperature conditions on fatigue behaviour of metal–polymer friction stir composite joints is available, indicating a gap in this field of knowledge. Considering the significant differences in thermo-mechanical behaviour between metals and polymers, it is expected that service temperature may affect significantly the performance of dissimilar friction stir composite joints in both monotonic and cyclical loading. In this research study, an experimental procedure was carried out to explore the impact of different service temperatures on static and fatigue strength of composite joints manufactured with FSW-based technology. The experimental data was subjected to a comprehensive analysis that included the probabilistic stress-life (*p*-S-N) of the composite joints, comparing the obtained results with joints fabricated with alternative joining technologies available in the literature. Moreover, to further identify and characterize the failure mechanisms, Differential Scanning Calorimetry (DSC) and Spectrum Electron Microscopy (SEM) were also performed.

## 2. Materials and Methods

All the studied friction stir composite joints were fabricated using AA6082-T6 (Poly Lanema, Ovar, Portugal) aluminum alloy and Noryl^®^ GFN2 (PHT, Le Versoud, France), with 2- and 5-mm nominal thickness, respectively.

The A6082-T6 alloy exhibits the highest strength value in the 6xxx series, together with excellent corrosion resistance, and is commonly used for the manufacturing of machined components [30,31,32,33]. The detailed chemical composition of this aluminum alloy is summarized in Table 2.

Noryl^®^ GFN2 is a proprietary blend of poly (phenylene ether) (PPE) and high impact polystyrene (PS) in undisclosed proportion, reinforced with 20% weight of short glass fibre. It is formed by injection moulding at temperatures ranging between 299–327 °C and, although PPE is a thermoset and PS is an amorphous thermoplastic, they are completely miscible, resulting in an amorphous thermoplastic with uniform microstructure and properties. This compatibility is essential to achieve the combination of properties exhibited by this polymer, which includes, very low moisture absorption, high strength, hydrolytic stability, low warpage, and dimensional stability [35,36].

**Figure 1:**
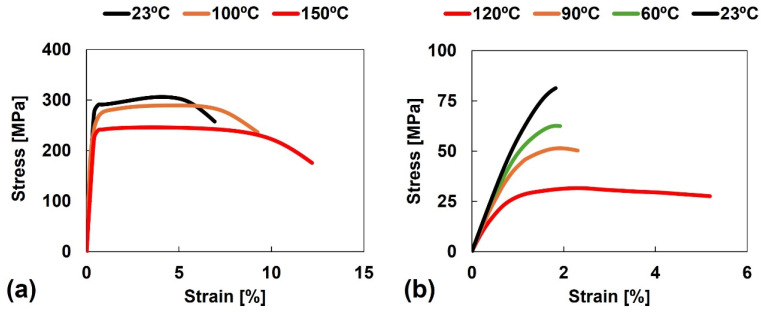
Engineering stress–strain curves under different temperatures of (**a**) AA6082-T6, adapted from [39], and (**b**) Noryl^®^ GFN2, adapted from [38].

The main thermo-mechanical and physical properties of these base materials are presented in Table 3, and the engineering stress-strain plots for different temperatures are depicted in Figure 1.

**Table 3:** Main physical and thermo-mechanical properties of AA6082-T6 [37] and Noryl GFN2 [38].

Base Material	ρ (g/cm^3^)	E (GPa)	$\sigma_{UTS}$ (MPa)	T_melt_ (°C)	T_g_ (°C)	K (W/(m°C))
AA6082-T6	2.70	70	>290	582	-	180
Noryl^®^ GFN2	1.25	6	80	280	135–145	0.26

*ρ*: density; E: Young’s modulus; *σ_UTS_*: mechanical strength; T_melt_: melting temperature; T_g_: glass transition temperature (softening onset); K: electrical conductivity.

**Figure 2:**
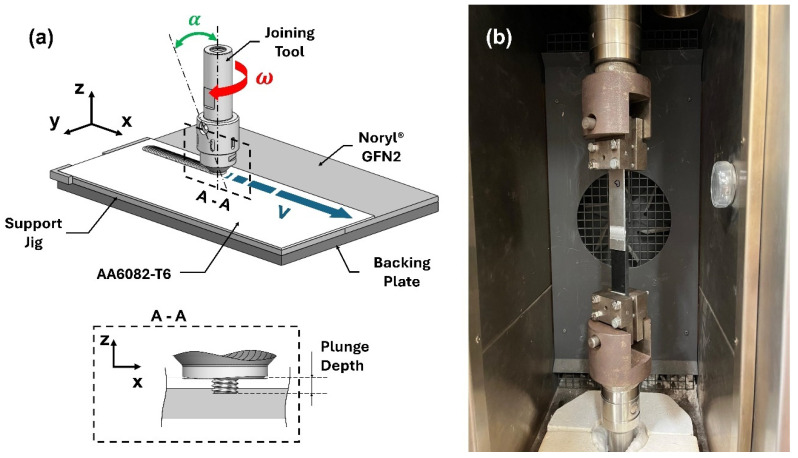
(**a**) Schematic of the fabrication setup with the main processing parameters (not to scale), and (**b**) specimens’ testing setup.

The joining process was thoroughly described elsewhere [40]. The aluminum plate was positioned on the retreating side and on top of the polymeric plate with 40 mm of overlapping length. The joining process was carried out under position control in a custom-built friction stir welding machine. The tool was composed by a flat scrolled shoulder (∅ 16 mm) and a threaded cylindrical probe (∅ 5 mm) (Figure 2). The main process parameters are listed in Table 4.

The resulting joints were machined perpendicularly to the joining path into 25 mm wide specimens. Specimens were subjected to quasi-static tensile shear testing using an electro-mechanical testing machine (Instron^TM^ 5566, Norwood, MA, USA). A 5 mm/min loading rate was applied, based on specification for tensile testing of plastics (ASTM D638), the weakest material in the joint. Fatigue tests were carried out in a servo-hydraulic testing machine (Instron^TM^ 8502), using a minimum/maximum load ratio, a frequency of 10 Hz and different load amplitudes, under load control. Both testing machines were coupled with a 10 kN loading cells and a temperature chamber (Instron^TM^ 3119-006), enabling testing under different temperatures: 23 °C (average room temperature), 75 °C, and 130 °C. These temperature values were established in previous research by the authors [40], and enable the study of joint behaviour from room temperature up to the onset of polymer softening (Table 3); the intermediate 75 °C values allowed to check for non-linear relationship between testing temperature and joint strength.

The fracture surface of tested specimens was observed by scanning electron microscopy (SEM, Hitachi^TM^ S2400, Tokyo, Japan) for microstructural characterization. For that purpose, polymer-based plates were carefully cut below the fracture surface resulting from mechanical testing, rendering samples approximately 15 mm height. Surfaces were then coated with Au–Pd alloy to prevent charge accumulation during observation. All images were acquired in secondary electrons mode.

Because of its high sensitivity to thermal history [36], Noryl^®^ GFN2 was also studied by differential scanning calorimetry (DSC, TA Instruments Q200, New Castle, DE, USA), to enlighten the effect of processing and service temperature on mechanical performance. Heating up to the processing temperature was first simulated; previous research [40] showed that joint temperature reaches 235 °C during friction stir. In as much, a first heating run took place where pristine Noryl^®^ GFN2 was heated up to 235 °C and cooled to room temperature, in air. At this point, the sample hold the same thermal history than Noryl^®^ GFN2 in the composite joint before mechanical testing. The same sample was then re-heated, to assess the effect of testing temperature (75 and 130 °C). To ensure reproducibility, three samples (9.1 ± 0.1 mg) were tested. In each run, the material was encapsulated in a covered aluminum pan and heated up to 235 °C at 10 °C/min, under nitrogen gas flow of 20 mL/min. An empty pan was used as reference; cooling took place in air. Background removal of the obtained DSC curves was carried out using the FITYK 0.9.8 freeware (https://fityk.nieto.pl/ accessed on 8 January 2025). Peak onset was identified using the first derivative test.

## 3. Results and Discussion

Visual inspection of the produced composite joints revealed a sound surface finish with well-defined onion rings and exit holes, and neglectable amount of flash. Specimens fabricated for tensile and fatigue testing exhibited consistent morphology along their cross section, characterized by a double concavity of extruded aluminum clinched into the polymeric base material. This morphology enabled the development of mechanical interlocking between base materials, acting as primary binding mechanism, as observed by Correia et al. in previous research [25,40].

### 3.1. Quasi-Static Strength Under Different Service Temperatures

**Figure 3:**
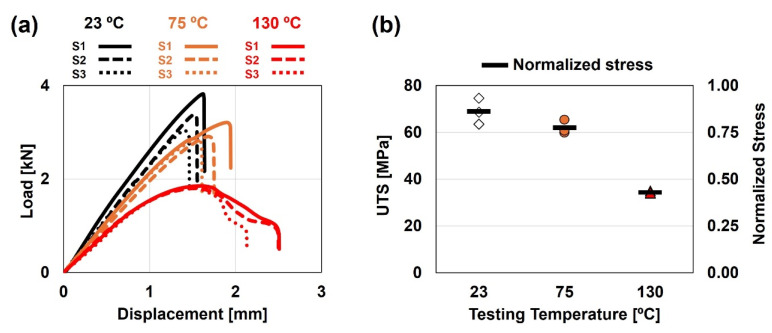
(**a**) Quasi-static tensile shear tests results at 23 °C, 75 °C, and 130 °C, and (**b**) joints’ UTS and corresponding normalized average stress.

The load–displacement curves obtained in quasi-static tensile shear tests were highly influenced by test temperature. An increase in test temperature induced a non-linear decrease of joints’ strength and stiffness, and a ductility increase (Figure 3a). The joints’ normalized stress (ratio between average joints’ UTS and UTS of Noryl at 23 °C) decreased from 86% at 23 °C, to 78% MPa at 75 °C, and 43% at 130 °C (Figure 3b). Joints’ strength decreased 10% and 50%, respectively, when tested at 75 and 130 °C, compared to room temperature. These results evidence the joints growing sensitivity to service temperature when approaching the softening temperature of Noryl^®^ GFN2, with good agreement with results by other authors [38,39].

All specimens failed at the polymer side of the joint, close to the interface, highlighting the strength of the binding mechanism within this region, regardless of service temperature. Failure apparently developed by crack nucleation in the transition region between the joining path and the low stiffness region, due to the development of secondary bending moment [25,40]. Development of a secondary bending moment is expected to result not only from the asymmetric joint geometry, with misalignment of neutral lines between both ends of the joint, but also due to the appreciable stiffness asymmetry between base materials. This induces a bending stress component, that stacks to the quasi-static tensile stress component throughout the quasi-static tensile shear test. Consequently, local stress at the failure region can be estimated according to the superposition principle [12,25] (Equation (1)):

(1) $$\sigma_{local}\approx \sigma_{tensile}+\sigma_{SBM}$$

where $\sigma_{local}$, $\sigma_{tensile},$ and $\sigma_{SBM}$ stand for local stress, tensile stress, and the stress resulting from the secondary bending moment at nucleation site, respectively.

This suggests that the mechanical behaviour of the joints is mainly governed and limited by the mechanical properties of the polymeric base material (the weakest part of the joint). Noryl^®^ GFN2 evidences a decrease in mechanical strength and stiffness (with slight increase in ductility) at temperatures below 90 °C, whereas at 120 °C, the material displays a significant drop in mechanical strength and stiffness (with notable increase in ductility) (Figure 1b).

Despite the lack of comparable data in the literature regarding dissimilar metal–polymer joints loaded above room temperature, a qualitative comparison with competitor technologies, such as adhesive bonding, can be established.

Viana et al. [41] studied the effect of different humidity and temperature values when testing bonded 6082-T6 alloy double cantilever beams using two different epoxy adhesives. At room temperature and humidity conditions, the adhesives displayed T_g_ close to 100 °C and to 120 °C. All specimen suffered adhesive failure when subjected to tensile shear testing at 80 °C. Vargas-Arista et al. [42] studied the mechanical behaviour of similar and dissimilar Dual Phase Steel and AA6061-T6 adhesively bonded tensile tested under different service temperature values. The dissimilar joint resulted in the lowest strength value, suffering adhesive failure. Apparently, mechanical performance of the adhesive at high temperature significantly degraded compared to results achieved at room temperature.

Both works concluded that the performance of adhesive bonded joints is very limited by the selected adhesive and to its response above room temperature. This further supports the importance of the results attained in the current study.

### 3.2. Fatigue Strength Under Different Service Temperatures

The influence of service temperature on fatigue life of friction stir composite joints was assessed by performing cyclic controlled loading with constant stress ratio and frequency, 0.1 and 10 Hz, respectively.

**Figure 4:**
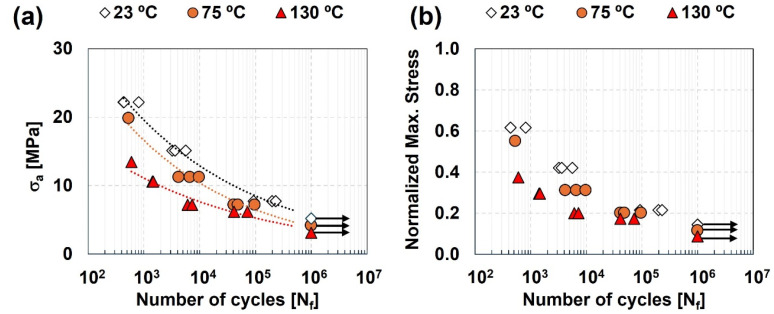
Plots of (**a**) stress amplitude vs. life with trend lines, and (**b**) normalized maximum cyclic stress vs. life. Black arrows denote runout tests at 10^6^ cycles.

Figure 4a displays attained fatigue curves for the tested temperatures, showing a downwards shift of the curves with increasing test temperature. The highest and lowest fatigue strength values were observed for test at 23 °C and 130 °C, respectively. Fatigue life values between 10^2^ and 10^5^ cycles were obtained for stress amplitudes that ranged between 7.7 and 22.2 MPa at 23 °C, between 7.2 and 19.8 MPa at 75 °C, and between 6.2 and 13.5 MPa at 130 °C. Infinite life ($N_{f}\geq 10^{6}\;cycles$) was obtained for stress amplitudes of 5.2, 4.2 and 3.1 MPa at 23, 75, and 130 °C, respectively.

To understand how the obtained stress values relate with room temperature UTS of Noryl GFN2, fatigue life values were also analyzed as a function of the normalized maximum cyclic stress (ratio of maximum applied cyclic stress and the UTS of Noryl^®^ GFN2 at 23 °C) (Figure 4b). Values of fatigue life between 10^2^ and 10^5^ cycles were obtained when submitting the specimens to normalized stress ranging from 0.24 to 0.63 at room temperature. Those values decreased to between 0.23 to 0.55 at 75 °C, and between 0.21 and 0.35 at 130 °C. All tested specimens failed at the polymeric side of the joints, close to the interface region, similarly to quasi-static tests results. This suggests that the fatigue behaviour of the joints is also governed and limited by the polymeric material.

The S–N curves were obtained by fitting a power law regression line to each $\sigma_{a}$ vs. N plot in Figure 4a. This way, considering $\sigma_{a}$ as an independent variable, the Basquin relationship with N_f_ can be established by Equation (2):

(2) $$\sigma_{a}=A\cdot {N_{f}}^{b}$$

where A and b are the adjusting parameters that maximize the fit of the regression curve fit to data points. The best fitting parameters for each testing temperature are listed in Table 5.

The coefficient of determination is higher than 0.9 for curves obtained at all tested temperatures. This suggests a good fitting of the regression curves to experimental data, especially regarding results obtained at 23 °C (0.980) and 75 °C (0.971).

**Figure 5:**
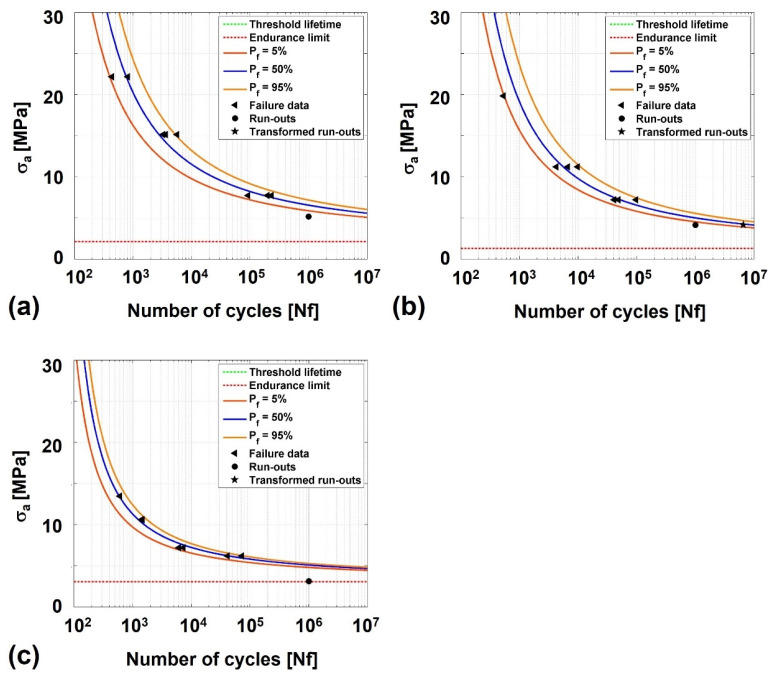
Probabilistic S–N curves for probabilities of failure of 5, 50, and 95% when the specimens are tested at (**a**) 23 °C, (**b**) 75 °C, and (**c**) 130 °C. Threshold lifetime below 10^2^ cycles is not displayed.

Results attained for the studied joints under cyclic loading at different service temperatures were compared to those of reinforced polymer–matrix composites under similar conditions reported in the literature. Mura et al. [43] studied the fatigue behaviour of ABS and PC-ABS thermoplastics at −27, 22 and 85 °C. They found that, compared to values at room temperature, the fatigue limit decreased around 10% in PC-ABS and around 50% in ABS when test temperature increases to 85 °C. Inasmuch as the results obtained in the current work are in line with reports in the literature [28,43], they emphasize the negative effect of high service temperature on performance, especially for values close to softening temperature of the polymeric base material. Furthermore, the uncertainty associated to the S–N regression curves due to scattering of experimental data was addressed by deriving the probabilistic S–N curves (p–S–N) using the Weibull distribution model [44,45,46,47,48]. Based on 3P Weibull distribution, Castillo and Fernández-Canteli [49] proposed the probabilistic stress-life model in Equation (3):

(3) $$F\left(\log\left(N_{f}\right);\log\left(∆\sigma \right)\right)=p=1-e^{{-\left(\frac{\left(\log\left(N_{f}\right)-B\right)\cdot \left(\log\left(∆\sigma \right)-C\right)-\lambda }{\delta }\right)}^{\beta }}$$

where

(4) $$\left(\log\left(N_{f}\right)-B\right)\cdot \left(\log\left(∆\sigma \right)-C\right)-\lambda \geq 0$$

where ∆σ is the stress range, F() is the cumulative probability of N_f_ associated to a ∆σ level, and the variables B and C are determined as follows:

(5) $$B=\log\left(N_{0}\right)$$

(6) $$C=\log\left(∆\sigma_{0}\right)$$

where $N_{0}$ and $∆\sigma_{0}$ are the threshold value of lifetime and the endurance fatigue limit, respectively. To improve the fit of the Weibull distribution to scattered data, the model incorporates three non-dimensional parameters: shape (*β*), scale (*δ*), and location (*λ).* These parameters, and the associated p–S–N curves for each testing temperature were computed using the ProFatigue^®^ software [50]. The Weibull parameters for each service temperature are listed in Table 6, and the corresponding *p*–S–N curves for 5%, 50%, and 95% probability of failure are displayed in Figure 5.

Depending on safety criticality of the joints, the designer should opt for the S–N curve corresponding to the given probability of failure. Additionally, given the brittle nature of the joints in the 23 to 75 °C range (please refer to Section 2), careful selection criteria are advisable, further emphasizing the need to determine the probabilistic S–N curves for metal–polymer friction stir composite joints.

### 3.3. Fracture Surface

**Figure 6:**
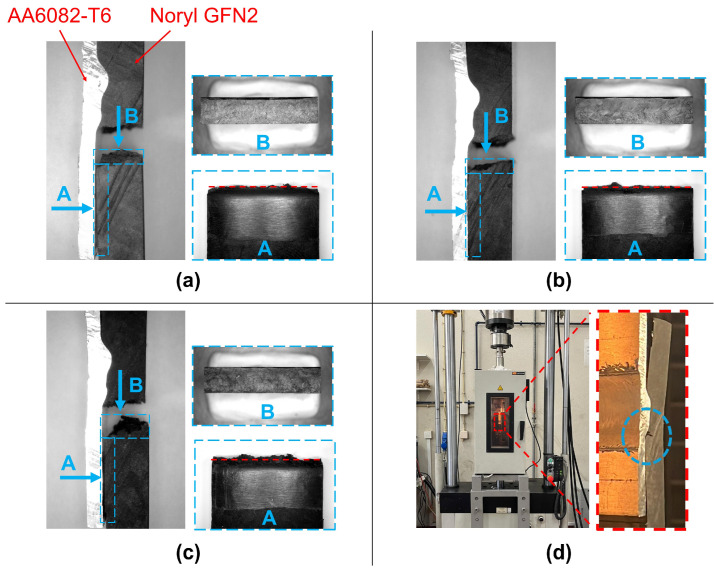
Macrographs of fracture surface after testing at (**a**) 23 °C, (**b**) 75 (°C), and (**c**) 130 °C; (**d**) detail of crack propagation on a specimen tested at 130 °C.

The fatigue specimens tested at 23 °C exhibited almost linear behaviour up to the failure load (Figure 3a), with limited amount of plastic strain and instantaneous crack propagation across the polymeric plate, as previously observed by Correia et al. [25,40]. Macroscopic observation of fracture surfaces apparently confirms the brittle nature of the joints at room temperature (e.g., Figure 6a, showing an almost flat bright surface).

Conversely, specimens tested at 130 °C exhibit larger amount of fracture displacement (Figure 3a), which indicates an increase in ductility. In this case, the joint failure was not instantaneous, and crack propagation is observable (Figure 6d). Also, fracture surface appears to be rough, with irregular dull surface (Figure 6c). Finally, specimens tested at 75 °C exhibited intermediate behaviour, and fracture surface with mixed features of both at 23 °C and 130 °C (Figure 6b).

**Figure 7:**
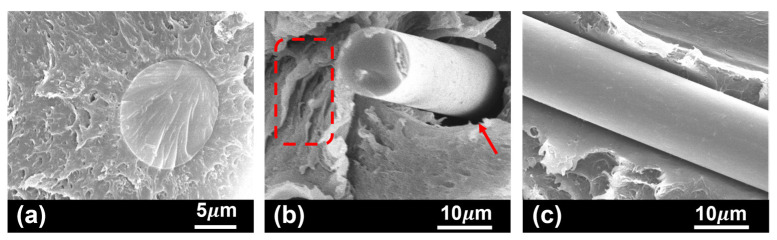
Most frequent toughening mechanisms identified in tested Noryl GFN2 samples: (**a**) strong fibre–matrix interaction, supporting stress transfer at the interface and fibre fracture (sample tested at room temperature); (**b**) polymer crazing (dashed region) and fibre pull out from the polymer matrix (arrow) (sample tested at 130 °C); and (**c**) fibre debonding from the polymer matrix (sample tested at room temperature).

Microstructural observation of the polymeric base material (Figure 7a) suggests a strong fibre/matrix interface. Interaction and adhesion of the polymer blend in Noryl to the glass fibres is totally supported by PPE, via hydrogen bonding with polar moieties at the glass surface [35]. The strong interfacial bond established is essential to high mechanical performance, allowing efficient stress transfer through the continuous polymeric matrix to the reinforcing phase [35]. In tested samples, several toughening mechanisms for energy dissipation to prevent rapid failure were identified. Regardless of test temperature, all studied samples evidenced some degree of deformation and crazing (Figure 7b), the most frequent energy dissipation processes in thermoplastics [51,52]. Mechanisms associated to fibres’ presence were also identified, including fibre pull out (Figure 7b), and fibre/matrix debonding (Figure 7c).

**Figure 8:**
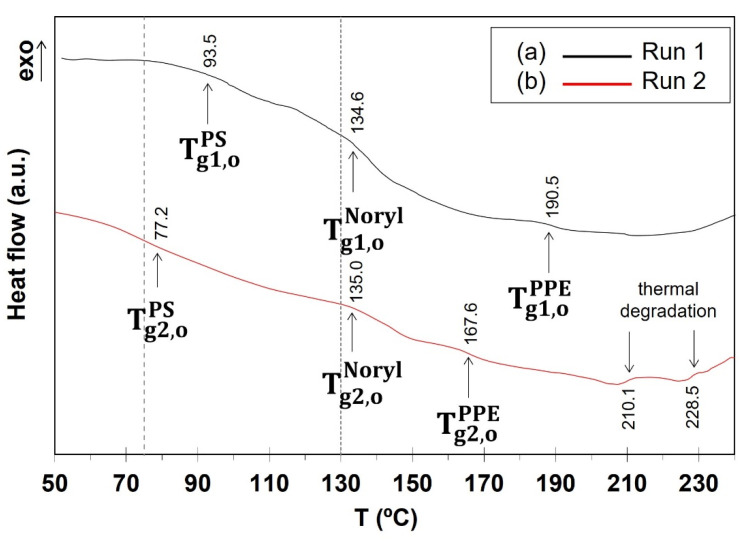
DSC curves of as-received Noryl (10 °C/min): (a) first heating run up to the joining temperature (235 °C), and (b) second heating run through the temperatures of mechanical testing (dashed lines). T_g1,o_: temperature of glass transition onset of a given phase on the first heating run; T_g2,o_: temperature of glass transition onset of the same phase on reheating.

**Figure 9:**
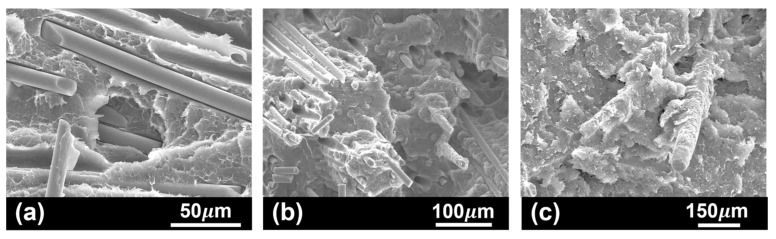
Low magnification images of the fracture surface of Noryl plates after mechanical testing at (**a**) room temperature, (**b**) 75 °C, and (**c**) 130 °C, showing fibre interaction with increasingly disordered matrix.

Thermal analysis results showed that heating as-received Noryl GFN2 up to the joining process temperature (235 °C) triggers three endothermal transitions (Figure 8a). The corresponding onset temperature values are consonant with glass transition temperature of a PS-rich phase ($T_{g1,o}^{PS}$ = 93.5 °C), of Noryl^®^ GFN2 ($T_{g1,o}^{Noryl}$ = 134.6 °C), and of a PPE-rich phase ($T_{g1,o}^{PPE}$ = 190.5 °C) [36]. Although the peaks corresponding to PS- and PPE-rich phases are very weak, their presence suggests that localized phase separation took place in the polymeric base material during the previous heating cycle [53], indicating fast and/or uneven cooling from the melt after injection moulding [35,54]. Importantly, testing Noryl^®^ up to 75 °C (Figure 9b) brings the material above glass transition onset of the PS-rich phase, while 130 °C (Figure 9c) surpass T_g_ of both the PS-rich phase and Noryl. Thus, while testing at room temperature is carried out upon a rigid solid (Figure 9a), testing at the higher temperatures is carried out upon a polymer which is undergoing increasing viscous flow [53] (Figure 1b). Polymer chains are increasingly allowed to move relative to each other, especially at 130 °C, while deformation imposed by the test causes increasing structural disarray, in good agreement with the increasingly disordered solidification structure displayed around fibres in Figure 9a–c.

At each tested temperature, the behaviour and fracture surface of specimens submitted to quasi-static test are in good agreement with those resulting from fatigue test. Furthermore, they are similar to Noryl’s behaviour at the same temperature, confirming that the joint is limited by the mechanical behaviour of the polymeric–matrix composite material.

## 4. Conclusions

The present research work was designed to analyze the effects of different service temperatures on mechanical behaviour of metal–polymer friction stir composites, both under quasi-static and cyclic loading conditions. With the experimental results it was possible to retrieve the following conclusions:

- In all tested scenarios, the failure of joints occurred at the polymeric base material close to the interface region, confirming the quality and strength of the binding mechanisms within this region.
- The joints mimic and are limited by the mechanical behaviour of the polymeric base material, regardless the testing conditions.
- Joints sensitivity to service temperature increases as it gets close to the softening temperature of Noryl^®^ GFN2, both under quasi-static and cyclic loading conditions.
- The quasi-static strength decreased by 10% and 56% when the testing temperature is increased to 75 and 130 °C, respectively.
- Fatigue lifespans over 10^4^ cycles were obtained with stress levels below 20 MPa (25% of the polymeric base material’s UTS at 23 °C), regardless of the testing temperature.
- At high temperatures, the polymeric base material undergoes softening and glass fibres debonding from the polymeric matrix, decreasing its load bearing capacity.
- At testing temperatures close to the softening temperature of Noryl^®^ GFN2, the joints show more ductility, and the failure occurs progressively, whereas at temperatures between 23 and 75 °C, the joints evidence a brittle nature with crack nucleation and instantaneous propagation.

Given the present joining technology’s early stage of use, there are no available design standards nor good practices applicable to metal–polymer friction stir composite joints. This way, the derived p–S–N curves for each service temperature are of useful interest when designing a component for a given purpose. For instance, when looking for performance, the component design process might consider the S–N curve for 95% probability of failure, while safe applications should consider the 5% curve.

## Figures and Tables

**Table 1:** Examples of friction stir based metal–polymer joints reported in the literature.

Base Materials	Joint Configuration	Joint Strength (MPa)	Reference
AA7075, GFR * PEEK	Lap Joint	~60	[16]
AZ31, GFR */CFR ** PPS	Lap Joint	~28	[17]
AA6061, PC	Butt Joint	~15	[18]
AA6061, CFR ** PA66	Lap Joint	~25	[19]
AA5058, PMMA	Lap Joint	~46	[20]
AA6061, PEEK	Lap Joint	~20	[21]
AA5059, HDPE	Butt Joint	~10	[13]
AA5754, PMMA	T Joint	~50	[22]
AA5052, CFR ** PA6	Lap Joint	~64 ***	[23]
AA5182, CFR ** PP	Lap Joint	~47 ***	[24]
AA6082, Noryl^®^ GFN2	Lap Joint	~70	[25]

* GFR—Glass Fibre Reinforced, ** CFR—Carbon Fibre Reinforced, *** Estimation based on maximum UTL and cross-sectional area of the polymeric base material.

**Table 2:** Main alloying elements of the parent AA6082 alloy [34].

Cr (%)	Fe (%)	Mg (%)	Mn (%)	Si (%)	Ti (%)	Zn (%)	Cu (%)	Al (%)
≤0.10	0.20–0.21	0.63–0.64	0.47–0.48	0.97–1.00	≤0.10	≤0.10	≤0.10	Balance

**Table 4:** Process parameters used in friction stir welding of the studied composite joints.

Dwell Time (s)	ω (RPM)	*v* (mm/s)	α (°)	Pin Length (mm)	Plunge Depth (mm)
15	1000	2.33	2	2	2.2

ω: rotational speed; *v*: travel speed; α: tilt angle.

**Table 5:** Parameters of power law regression equations and coefficients of determination.

Temperature (°C)	A	b	R^2^
23	68.383	−0.181	0.980
75	68.096	−0.204	0.971
130	34.042	−0.162	0.916

A: scaling factor; b: power law exponent; R^2^: coefficient of determination.

**Table 6:** Weibull parameters for probabilistic modelling of S–N curves at each service temperature.

Temperature (°C)	β	δ	λ	B	C
23	5.19	4.12	11.72	0 (1 cycle)	0.76 (2.13 MPa)
75	3.18	2.78	16.03	0 (1 cycle)	0.27 (1.31 MPa)
130	2.46	5.62	0.30	2.46 (11 cycles)	1.11 (3.05 MPa)

β: shape parameter; δ: scale parameter; λ: shift parameter; B: location parameter related to the cycle-life behaviour; C: location parameter related to stress.

## Data Availability

The original contributions presented in this study are included in the article. Further inquiries can be directed to the corresponding author.
